# Correction: Stalder, et al.; Value of SUV_max_ for the Prediction of Bone Invasion in Oral Squamous Cell Carcinoma. *Biology* 2020, *9*, 23

**DOI:** 10.3390/biology9060113

**Published:** 2020-05-29

**Authors:** Stephanie A. Stalder, Paul Schumann, Martin Lanzer, Martin W. Hüllner, Niels J. Rupp, Martina A. Broglie, Grégoire B. Morand

**Affiliations:** 1 Department of Otorhinolaryngology - Head and Neck Surgery, University Hospital Zurich, 8091 Zurich, Switzerland; stephistalder@gmx.ch (S.A.S.); martina.brogliedaeppen@usz.ch (M.A.B.); 2 Faculty of Medicine, University of Zurich, 8006 Zurich, Switzerland; paul.schumann@usz.ch (P.S.); martin.lanzer@usz.ch (M.L.); martin.huellner@usz.ch (M.W.H.); niels.rupp@usz.ch (N.J.R.); 3 Department of Cranio-Maxillo-Facial and Oral Surgery, University Hospital Zurich, 8091 Zurich, Switzerland; 4 Department of Nuclear Medicine, University Hospital Zurich, 8091 Zurich, Switzerland; 5 Department of Pathology and Molecular Pathology, University Hospital Zurich, 8091 Zurich, Switzerland

The authors would like to make a correction to their published paper [1].

The authors would like to change one incorrect *p*-value in paragraph 2.6 “Survival outcomes” and the corresponding Figure 4. The correct *p*-value for the log-rank test for distant metastasis-free survival between patients with bone invasion vs. without bone invasion (Figure 4, Panel C) is 0.144 (and not 0.032). The correct *p*-value for the log-rank test for disease-specific survival (Figure 4, Panel D) is in turn 0.032 (and not 0.144). The change does not affect the scientific results. The rest of the manuscript does not need to be changed.

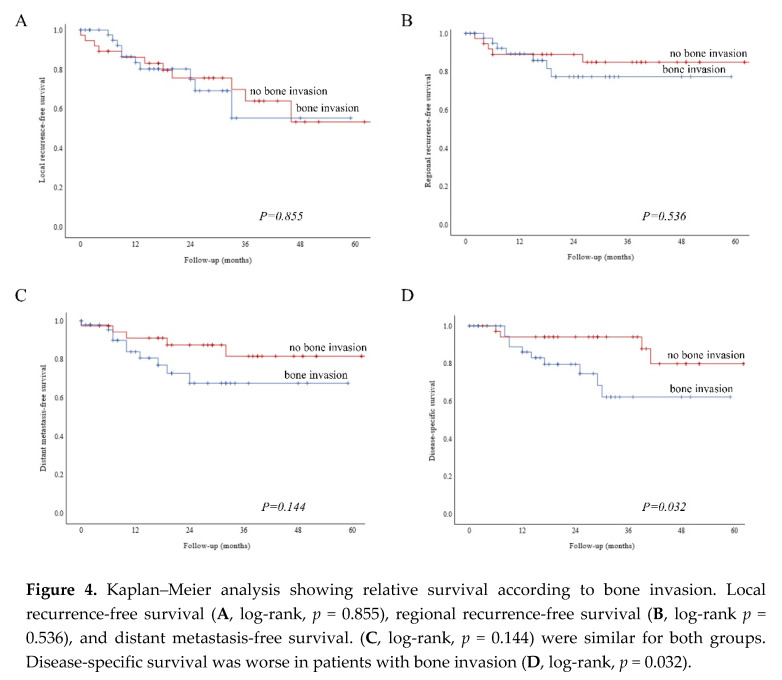



The authors would like to apologize for any inconvenience caused. The manuscript will be updated, and the original will remain available on the article webpage.
